# Insight into the Molecular Mechanism of Diabetic Kidney Disease and the Role of Metformin in Its Pathogenesis

**DOI:** 10.3390/ijms241713038

**Published:** 2023-08-22

**Authors:** Marcin Kleibert, Przemysław Zygmunciak, Klaudia Łakomska, Klaudia Mila, Wojciech Zgliczyński, Beata Mrozikiewicz-Rakowska

**Affiliations:** 1 Chair and Department of Experimental and Clinical Physiology, Laboratory of Centre for Preclinical Research, Medical University of Warsaw, 02-097 Warsaw, Poland; marcin.kleibert@gmail.com; 2 Faculty of Medicine, Medical University of Warsaw, 02-091 Warsaw, Poland; zygmunciakprzemyslaw@gmail.com (P.Z.); klaudyna.mila@gmail.com (K.M.); 3 Faculty of Medicine, Wroclaw Medical University, 50-367 Wroclaw, Poland; klaudia.lakomska@gmail.com; 4 Department of Endocrinology, Centre of Postgraduate Medical Education, Bielanski Hospital, 01-809 Warsaw, Poland; klinendo@bielanski.med.pl

**Keywords:** diabetic kidney disease, metformin, pathogenesis, polyol pathway, hexosamine pathway, PKC pathway, AGE pathway

## Abstract

Diabetic kidney disease (DKD) is one of the leading causes of death among patients diagnosed with diabetes mellitus. Despite the growing knowledge about the pathogenesis of DKD, we still do not have effective direct pharmacotherapy. Accurate blood sugar control is essential in slowing down DKD. It seems that metformin has a positive impact on kidneys and this effect is not only mediated by its hypoglycemic action, but also by direct molecular regulation of pathways involved in DKD. The molecular mechanism of DKD is complex and we can distinguish polyol, hexosamine, PKC, and AGE pathways which play key roles in the development and progression of this disease. Each of these pathways is overactivated in a hyperglycemic environment and it seems that most of them may be regulated by metformin. In this article, we summarize the knowledge about DKD pathogenesis and the potential mechanism of the nephroprotective effect of metformin. Additionally, we describe the impact of metformin on glomerular endothelial cells and podocytes, which are harmed in DKD.

## 1. Introduction

Metformin is a guanide derivative (dimethylbiguanide) used in various pathologies, among which type 2 diabetes mellitus (T2DM) is one of the most prominent. Despite the results of in vivo studies conducted in 1929 which showed the blood-sugar-lowering properties of the biguanides, it was not till the late 1950s that metformin became well-recognized as an antidiabetic agent. It was introduced to treat diabetes in Europe in 1958 [1]. Since then, much has been done to further clarify the mechanism of action of the medicine. The blood-sugar-lowering activity seems to be mostly caused by the reduction in hepatic gluconeogenesis and increased peripheral glucose uptake due to increased receptor sensitivity, but other mechanisms, including elevating β-cells function and microbiota modulation, are also proposed [2].

Metformin not only successfully decreases hyperglycemia, but also stabilizes the weight of some patients, may act favorably on lipid profile, and increases the survival of diabetic patients mostly by reducing the risk of cardiovascular events. Considering all of the mentioned benefits, it became the World Health Organization’s essential medicine. Several clinical trials have confirmed the effectiveness of metformin. The United Kingdom Prospective Diabetes Study (UKPDS) was one of the first randomized clinical trials to demonstrate the effectiveness of metformin use on diabetic complications in T2DM [3]. The authors confirmed in 10-year follow-up that the intensive metformin therapy can cause risk reduction of 33% in myocardial infarction, 20% in stroke, and 16% in microvascular complications, defined as vitreous hemorrhaging, retinal photocoagulation, or renal failure in patients diagnosed with T2DM [4]. In another study, it was shown that a switch from glibenclamide to metformin resulted in significant reduction in the urine albumin secretion by a mean of 24.2 mg/day during 12-week follow-up [5]. Kwon et al. showed in a retrospective study (10426 T2DM individuals with CKD stage 3) that long-term metformin use was associated with 33% (HR 0.67; 95% CI 0.58–0.77) risk reductions in ESRD progression, respectively (median follow-up period: 7.3 ± 4.8 years) [6]. However, in a few studies, it was shown that metformin does not prevent albumin-to-creatinine ratio (ACR) increment as effectively as rosiglitazone and glyburide [7].

The recent ADA/EASD consensus diminished the role of metformin in the treatment of T2DM patients [8].

Metformin is still favored when the main therapy goal is glycemic management [8]. Although, among high-risk patients, where cardiorenal risk reduction is the primary target, sodium-glucose cotransporter 2 inhibitors (SGLT2i) and glucagon-like peptide 1 receptor agonists (GLP-1 RA) are preferred [8]. Metformin can also be used in prediabetic patient groups, women that suffered from gestational diabetes and young patients with a high risk of developing T2DM, as a preventive measure [9]. In addition, it plays a role in the management of insulin-resistant pathologies such as polycystic ovarian syndrome (PCOS) [10] and HIV-associated lipodystrophy [11]. Interestingly, some studies have also shown its tumor-protective action, which might become an interesting novel field of research [12,13].

As our awareness of molecular mechanisms of metformin rises, more and more previously unknown possible indications of metformin are elicited. Recently, the interest of scientists was drawn to the benign yet important pleiotropic effect of metformin on the kidney. In particular, the influence of medicine on diabetic kidney disease (DKD) is being investigated. In this review, we would like to describe recent advantages in the understanding of molecular mechanisms of DKD and the significance of metformin in diabetic nephropathy.

## 2. Review

### 2.1. Diabetic Kidney Disease—An Overview

Around 40% of T2DM patients will develop chronic kidney disease (CKD) during their lives [14]. Moreover, 10% of T2DM patients will die because of renal failure, making renal complications an important point in diabetic care [15]. DKD is characterized by an increased urinary albumin-to-creatinine ratio > 3 mg/mmol over a period of time longer than 90 days and/or a persistent reduction in eGFR to <60 mL/min/1.73 m^2^ [16]. The metabolic dysregulation drives the cellular changes of DKD that are particularly distinct in the podocytes’ and glomerular endothelial cells’ populations [17]. With the progression of the disease, morphological changes in the kidney, such as thickening of the glomerular, capillary, and tubular basement membrane; loss of endothelial fenestrations; mesangial matrix expansion; and loss of the podocytes, are to be found [18]. Despite the distinctive microscopic image, biopsy of the kidney is not generally needed to diagnose the condition, especially for patients that suffer from diabetes for a long time and do not have atypical findings (e.g., rapid onset of proteinuria, rapid progression, and absent retinopathy) [19]. Although, it is noteworthy that renal biopsy remains a gold standard of obtaining diagnostic and prognostic information in DKD [20].

The development of DKD is associated with both hereditary and environmental factors [21,22]. While the most prominent established risk factors of the disease remain hyperglycemia and hypertension [18], the narrow sense of the heritability of DKD is estimated to be 35% [23]. Indeed, genetic variants associated with DKD for both type 1 (e.g., AFF3, RGMA/MCTP2 [24]; UMOD [25]) and type 2 diabetes mellitus (e.g., SCAF8/CNKSR3, MYH9 [26]; UMOD [25]) are suggested. A recent study performed by Sheng et al. shed new light on the subject by performing an integrative genetic and epigenetic analysis. The group distinguished four DKD phenotypic manifestations (glycemia, albuminuria, kidney function, and kidney function decline) and connected them to the specific methylation changes. Interestingly, DKD phenotypes considerably overlapped with glycemia-associated methylation, which might suggest the big role of glycemia in other traits [22].

Indeed, hyperglycemia and glucose control have a huge impact on the development of DKD. Poor diabetes management translates into a higher risk of nephropathy, even despite years of great glycemic control [27]. The mechanism behind this phenomenon called “glycemic memory” remains unclear and probably it is an interplay between several alterations such as epigenetic changes, increased production of reactive oxygen species (ROS), accumulation of advanced glycation end products (AGEs), and others [28,29]. Increased intercellular level of glucose activates several metabolic pathways (namely polyol pathway, hexosamine pathway, protein kinase C (PKC) pathway, AGE pathway, and Rho-associated coiled-coil containing protein kinase (ROCK) pathway), which results in redox imbalance that is a starting point for DKD progression [30,31] (Table 1). Interestingly, most of them can be regulated by metformin. It was shown that metformin therapy can reduce the oxidative stress induced by the overactivation of these pathways.

### 2.2. Molecular Pathways Involved in Pathogenesis of DKD

#### 2.2.1. Polyol Pathway–Overview and Impact of Metformin

Increased sugar level elevates the amount of glucose being metabolized by the polyol pathway (Table 1). This mechanism has two steps: firstly, glucose is converted by aldose reductase (AR) to sorbitol, which is oxidized by sorbitol dehydrogenase (SDH) to fructose. The pathway requires NADPH and NAD+ as the enzyme cofactors [30,32]. Interestingly, the AR content of diabetic patients is increased in comparison to non-diabetic patients, which shows the importance of this pathway in the diabetic kidney [33]. The prevailing theory states that the polyol pathway damages the kidney through the increased usage of NADPH, which is also necessary to oxidize reduced glutathione that is an ROS scavenger [32]. What is more, the intermediate product of this pathway—sorbitol—is highly hydrophilic and its intracellular buildup might result in osmotic damage to the cell [34]. This pathway is involved in the development of DKD. It seems that, by inhibiting this pathway, the development of DKD could be delayed. Based on studies, the activity of the polyol pathway in the kidney is very high [35,36]. Indeed, it was shown that the concentration of key enzyme aldose reductase (AR) is the highest in the kidney medulla (29.3 microgram/milligram protein), followed by sciatic nerve (5.1 microgram/milligram protein) [37]. This leads to more pronounced oxidative and osmotic stress in the kidneys.

There is not enough related to the impact of metformin impact on the activity polyol pathway in kidneys. However, Jin Lee, Hae-In Lee, et al. showed on non-obese type 2 diabetic mice (STZ injection) that metformin can decrease histological changes in kidneys which are caused by hyperglycemia. In addition, they observed that, in diabetic mice, the activity of aldolase reductase (AR) was increased by 17% (*p* > 0.05) in comparison to the control group. This effect was diminished by metformin treatment. These observations suggest that the polyol pathway can play an important role in DKD progression and metformin can reduce its negative impact on the progression of this complication of DM [38].

Based on a study with a rodent model of T2DM, the other authors checked the oxidative stress in the kidney. Levels of LO (149.7%) and PO (16.3%) were higher and levels of renal GSH (38.1%) were lower in high-fat diet animals compared to control groups (*p* < 0.05 for all comparisons) [39]. The previously mentioned results may be associated with high activity of the polyol pathway and metformin treatment may prevent these changes [39,40].

#### 2.2.2. Hexosamine Pathway—Overview and Impact of Metformin

Another enzymatic pathway that contributes to the development of diabetic complications is the hexosamine pathway (Table 1), in which fructose 6-phosphate is converted by glutamine:fructose 6 phosphate (GFAT) to uridine diphosphate-N-acetylglucosamine (UDP-GlcNAc). UDP-GlcNAc is a donor of glycosyl chains that, among others, are being used to modify nuclear proteins [29,31]. As a result, induction of transforming growth factor β1 (TGF-β1) is to be expected in all epithelia, including renal tubuli [40,41]. TGF-β plays a pivotal role in renal fibrosis and the progression of DKD [42].

In the normoglycemic environment, almost all glucose from cells is metabolized through glycolysis. However, 2% to 5% take part in the hexosamine pathway. It seems that the rate-limiting enzyme is glutamine:fructose-6-phosphate-amidotransferase (GFAT), which regulates the amount of glucose in this pathway. The negative effect of high glucose levels may be partially diminished by the inhibition of GFAT. The exposure to 30 mmol/L D-glucose promotes TGF-β production in mesangial cells [41]. Moreover, the TGF-β1 concentration is higher among diabetic patients in comparison to the control group (29.84 ± 7.04 ng/mL vs. 11.37 ± 4.06 ng/mL, *p* < 0.001) [43]. There is no direct evidence that metformin may regulate the hexosamine pathway. Yener et al. showed that metformin therapy does not reduce the serum concentration of TGF-β1 among patients with DM [43]. However, it was shown that the metformin inhibits TGF-β1 binding to the receptor, which results in decreased downstream signaling [44]. It may be associated with a protective effect against renal fibrosis but this mechanism is not directly associated with the hexosamine pathway.

#### 2.2.3. PKC Pathway—Overview and Impact of Metformin

Elevated PKC activation (Table 1) is caused mainly by increased conversion of DHAP to glycerol-3-phosphate that generates 1,2-diacylglycerol (DAG), which acts as a PKC stimulator [30], but other mechanisms such as activation by stable analogs of thromboxane A2/prostaglandin endoperoxide are also known [41,42]. PKC can cause damage in kidneys by phosphorylating subunits of NADPH oxidase, which leads to excessive ROS production [30]. It might also play a role in decreasing nitric oxide (NO) production, which is associated with the development of vascular complications [43,44].

It has been found that the inactivation of PKC isoforms by its specific inhibitors reverses many diabetes- or hyperglycemia-associated vascular dysfunctions, including those which develop in kidneys [45]. Presently, the PKC family contains 13 isoforms, which can be classified into three subfamilies based on their mode of activation: the group of classical or conventional PKC (cPKC) consists of the α, βI, βII, and γ isoforms (activated by calcium (Ca), DAG or its analog phorbol 12-myristate 13-acetate (PMA), and phosphatidylserine (PS)), the new PKC (nPKC) includes the subtypes δ, ε, η, and θ isoforms (independent of Ca but require DAG and PS), and the third group of isoforms, the atypical PKC (aPKC) ξ, ι/λ, ν, and µ, activation of which does not require Ca or DAG but only PS [42,44].

As was mentioned, DKD is characterized by excessive accumulation of extracellular matrix (ECM), in which reactive oxygen species (ROS) play a central role in synthesis. High glucose level induces cellular ROS through PKC-dependent activation of NAD(P)H oxidase and through altered mitochondrial metabolism that further promotes the formation of advanced glycation end products and enhanced ECM synthesis and leads to renal fibrosis [42]. It seems that inhibition of PKC may reduce renal fibrosis and delay the development of DKD.

K.R. Tuttle et al. found that inhibition of PKC β may improve microvascular and macrovascular outcomes in diabetes [46].

Despite there being no studies about metformin’s impact on PKC in kidneys, its role in the PKC pathway was investigated in other tissues.

M. Mahrouf et al. examined whether metformin exerts its antioxidant effect through modulation of PKC activity in bovine aortic endothelial cells (BAEC). They showed that metformin decreased ROS production on a direct PKC activator (PMA), angiotensin II (ANG), and glucose-stimulated BAEC in a similar way to that obtained by PKC-specific inhibitors (calphostin C and chelerythrine) alone. On the other hand, metformin reduced PKC membrane translocation in ANG- and PMA-stimulated cells and reduced kinase activity in PMA-activated cells only. Finally, in vitro incubation with purified PKC indicated that metformin had no direct effect on PKC activity. However, it suggests that metformin exerts intracellular antioxidant properties by decreasing ROS production through the inhibition of PKC activity [47].

Similar conclusions were shown by A. Gallo et al. in their investigation of human umbilical vein endothelial cells. They demonstrated that in vitro metformin attenuates the translocation of PKC-β2 from the cytosol to the membrane, which was induced by hyperglycemia. Additionally, metformin therapy reduces hydrogen peroxide induction of PKC-β2 [48].

Furthermore, B. Batchuluun et al. showed (examination of human aortic endothelial cells) that translocation of p47phox (a regulatory subunit of NAD(P)H oxidase) induced by high glucose levels was reduced by metformin treatment. Similarly, NAD(P)H oxidase activity was increased in hyperglycemic conditions and inhibited by metformin. These findings were consistent with their previous report showing that high glucose level stimulates ROS production mainly through activation of the PKC-NAD(P)H pathway. They also found that a mutant PKCβ that lacks DAG/binding site was not translocated in response to high glucose stimulation but it was capable of translocating by another stimulus, histamine. This observation provides direct evidence that DAG might be necessary for high glucose-induced PKCβ activation. It was also shown that metformin may reduce total DAG levels and ROS production as a consequence [49].

The above research showed metformin’s positive impact on PKC-dependent pathways in endothelial cells. Nevertheless, more studies that will evaluate the impact of metformin in kidneys are needed. Moreover, there is a hope which should be checked in clinical trials that therapies targeting PKC isoforms may be used in delaying, stopping, and even reversing diabetic renal complications.

#### 2.2.4. AGE Pathway—Overview and Impact of Metformin

Hyperglycemia also induces nonenzymatic metabolic pathways that are important for DKD pathogenesis. In diabetic kidney, metabolites bind with glycating compounds extensively creating AGEs (Table 1) that localize primarily within the intima [50]. AGEs differ from their precursors and may interact not only with their receptors (receptor for AGE (RAGE)), but also with other compounds causing damage by modifying their functional domains [51]. Additionally, oxidative stress in diabetes results in the formation and accumulation of advanced glycation end products (AGEs) [52].

AGEs, largely via RAGE, activate signaling mechanisms that cause cell stress, contribute to cellular dysfunction, and damage target organs, including kidneys [53]. The formation is irreversible and, once formed, they continue to accumulate in tissues. AGE accumulation is one of the crucial factors in the progression of metabolic and pathophysiological mechanisms in DKD [54]. There is more and more evidence that anti-AGE strategies have an important role to play in the treatment of diabetic patients in the prevention of the progression of DKD [55].

S. Maeda et al. have shown that neutralizing antibodies against RAGE block the effects of AGEs on reactive oxygen species (ROS) generation via downregulation of RAGE expression [56].

Y. Ishibashi et al. examined the impact of metformin on suppressing reactive oxygen species (ROS) generation via reducing receptors for AGEs (RAGE) expression. They showed that the treatment of 0.01 and 0.1 mM metformin prevents AGEs-upregulated RAGE mRNA levels and subsequently increased ROS generation in human renal proximal tubular cells. They further examined that metformin reduced basal RAGE gene expression in tubular cells. Interestingly, 0.01 and 0.1 mM metformin gave the same inhibitory effect on RAGE mRNA levels. Moreover, compound C (an inhibitor of AMP-activated protein kinase) significantly blocked the beneficial effects of 0.1 mM metformin on RAGE mRNA levels and ROS generation in AGEs-exposed tubular cells, which suggests that metformin inhibited the AGEs-evoked inflammatory and fibrotic reactions in cells by reducing ROS generation via downregulation of RAGE expression through AMP-activated protein kinase activation. Next, they investigated whether metformin could block the AGEs-induced inflammatory and fibrotic reactions in tubular cells. Furthermore, metformin at 0.01 and 0.1 mM significantly inhibited the AGEs-induced upregulation of ICAM-1, MCP-1, and TGF-β mRNA levels in tubular cells. Metformin dose-dependently also blocked the AGEs-induced tubular cell apoptosis evaluated by DNA fragmentation in ELISA. Furthermore, cell surface fluorescence intensity of annexin V, another marker of apoptosis, was significantly decreased by the treatment with metformin; 1 mM metformin completely suppressed the proapoptotic effects of AGEs in tubular cells. In this study, they did not clarify the exact molecular mechanism of RAGE gene suppression by metformin. This fact can lead to further studies with very promising results in the future of exploring metformin molecular mechanisms [57]. Additionally, a better understanding of these mechanisms may result in discoveries of new therapeutic targets.

### 2.3. Glomerular Endothelial Cell (GEC) Dysfunction

#### 2.3.1. GEC Dysfunction in DKD—Overview

**Figure 1:**
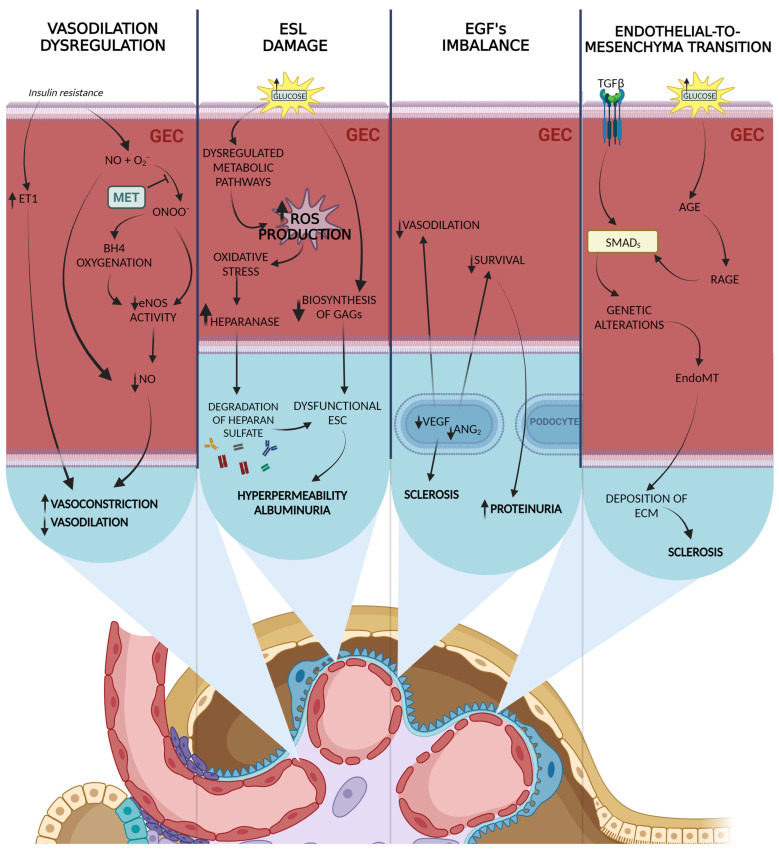
Main molecular pathways involved in GEC injury. The endothelium of diabetic patients differs from that of healthy subjects, exhibiting decreased endothelium-dependent vasodilation and altered signaling pathways. The decrease in vasodilating effects of nitric oxide (NO) may be caused by increased endothelin-1 (ET-1) signals and the formation of peroxynitrite (ONOO^−^) due to the reaction between NO and superoxide anion. Insulin resistance further contributes to a weaker vasodilator response and potential vasoconstriction. Diabetic glomerular endothelial cells (GECs) experience increased permeability and damage to the endothelial surface layer (ESL), primarily through oxidative-stress-induced heparanase production and reduced glycosaminoglycan biosynthesis. Imbalances in growth factors such as vascular endothelial growth factor (VEGF) and angiopoietins contribute to endothelial dysfunction and glomerulosclerosis in diabetic kidney disease. Additionally, endothelial-to-mesenchymal transition (EndoMT) plays a role in fibroblast accumulation and the progression of DKD. Transforming growth factor beta (TGFβ) and AGE/RAGE/Smad3 pathways are implicated in these processes. Created with Biorender.com.

All of the aforementioned pathways increase ROS within GECs by either their overproduction and/or decreased elimination (Figure 1). The major sites that are able to produce superoxide are localized within mitochondria. Hyperglycemia elevates the voltage gradient across the mitochondrial membranes, which translates to increased superoxide formation and increases ROS production [32,58,59]. Consistent with these observations, diabetes interferes with the mitochondrial life cycle, promoting the dominance of dysfunctional mitochondria which are characterized by increased ROS production in comparison to regular mitochondria [60,61]. What is more, in diabetes, both NADPH oxidase and aldose reductase system activity is increased, which leads to increased superoxide and superoxide anion formation [58,62]. These mechanisms increase the oxidative stress of a cell, which probably is a starting point for diabetic complications [58].

More and more studies suggest that GEC injury plays a major role in the development and progression of DKD. Adjustment of the glomerular endothelial cell surface layer, including glycocalyx, its major component, is a leading cause of microalbuminuria observed in early DKD. The glomerular filtration barrier (GFB) comprises an innermost fenestrated GEC layer, a glomerular basement membrane (GBM), and an outermost layer of podocytes with their interdigitating foot processes bridged by a slit diaphragm. Injury of any of these three components of the GFB will lead to albuminuria and the progression of DKD [63].

E. J. Weil et al. in their study of patients with type 2 diabetes mellitus found that both podocyte damage and glomerular endothelial injury were present in a cohort with macroalbuminuria but, interestingly, compared with podocyte injury, endothelial abnormalities were more closely associated with increasing urine albumin excretion. It suggests that GEC injury may be more critical to glomerular alterations in DKD than the commonly viewed importance of podocyte injury [64].

Interestingly, the endothelium of diabetic patients differs from that of healthy subjects. For example, endothelium-dependent vasodilatation among T2DM patients is decreased [65]. It might be partially caused by a metabolic switch that promotes endothelin-1 (ET-1) signals over vasodilating effects of NO, which involves previously described mechanisms. Thus, NO might react with superoxide anion, creating peroxynitrite (ONOO-), which decreased its bioavailability. ONOO- can reduce NO production by decreasing the catalytic activity of endothelial NO synthase (eNOS) [66]. It might also oxidize eNOS cofactor—tetrahydrobiopterin (BH4), which translates to eNOS uncoupling and further ROS production [67]. Insulin itself is also a stimulant of vasodilatation and its effect is dependent on endothelial NO production [68]. Thus, insulin resistance is associated with a weaker vasodilator response [69]. On the other hand, insulin may also stimulate endothelin production [70]. Altogether, insulin resistance might shift an endothelial response towards vasoconstriction, which may encourage endothelial damage.

Aside from deregulated vasodilatation, diabetic GECs are characterized by increased permeability, which is primarily caused by alterations within the endothelial surface layer (ESL). Increased oxidative stress leads to heparanase production, which results in ESL damage, mostly by the degradation of heparan sulfate proteoglycan [71]. Moreover, hyperglycemia decreases the biosynthesis of glycosaminoglycans [72]. Subsequently, these alterations drive hyperpermeability and albuminuria [71].

Another important point in GEC injury induction is the endothelial growth factors’ imbalance. In physiological conditions, podocytes steadily express low levels of vascular endothelial growth factor-A (VEGF-A) and angiopoietin-1 (Ang-1) that bind to receptors localized on GECs and podocytes [73]. However, hyperglycemia encourages alterations in both VEGF-A and angiopoietins activity [73]. VEGF-A is strongly overexpressed in the initial phase of the disease, with a slight downregulation with the progression of the condition [74,75]. This growth factor plays an important role in endothelial cell differentiation, proliferation, and survival, as well as facilitating endothelium-dependent vasodilatation and increasing vascular permeability. It seems that reduction in its concentration may result in severe proteinuria in DKD [76]. Among angiopoietins, an increase in both Ang-1 and Ang-2 is visible in the early phase, though, the more advanced the disease, the more decreased the Ang-1 level [77]. The said imbalance is correlated with DKD advancement characterized by many traits, such as proteinuria and glomerulosclerosis [78,79]. Additionally, it was shown that concentration of VEGF-D correlates with renal dysfunction, albuminuria, and proteinuria in patients with DKD [80].

Glomerulosclerosis might also be induced by a process called endothelial-to-mesenchymal transition (EndoMT), whereas endothelial cells stimulated by cytokines undergo alterations, leading to an acquisition of mesenchymal phenotype. Zeisberg et al. have found that above 30% of fibroblasts in chronic kidney disease (CKD) mouse models might derive from endothelium, since they expressed CD31, being a well-known endothelial marker, aside from fibroblasts and myofibroblasts markers [81]. This finding was later confirmed by using Tie1-Cre;R26R-stop-EYFP transgenic mice, on which endothelial lineage tracing was conducted [81]. Similarly, in diabetic kidneys, a great number of fibroblasts derive from endothelium [82]. The process of EndoMT is likely initiated by transforming growth factor beta (TGFβ) and contributes to the early stages of DKD progression [82]. Although, other pathways such as AGE/RAGE/Smad3 are of great importance in the initial stages of the transformation [83].

Lastly, it was reported that hyperglycemia results in leucine-rich α-2-glycoprotein 1 (LRG1) overexpression, which promotes angiogenesis and GEC injury by TGF-β/activin receptor-like kinase 1 (ALK1) pathway [84]. The deletion of LRG1 diminishes the deteriorating effects of hyperglycemia, such as albuminuria, endothelial injury, and podocyte damage [84]. What is more, these renoprotective actions are maintained long term and the deletion is not associated with worsened physiological outcomes in non-diabetic mice; therefore, an intervention within this pathway might be a novel potential target for attenuating DKD progression [84].

#### 2.3.2. Impact of Metformin on GEC

As was mentioned, in GECs, hyperglycemia leads to saturation of glucose metabolism and results in the activation of deleterious pathways such as the polyol pathway, the hexosamine pathway, the AGE/RAGE axis, and the PKC pathway, leading to endogenous ROS overproduction [17]. Progressive DKD is also associated with a deficiency of NO, uncoupling of eNOS in GECs, and the formation of superoxide and peroxynitrite (ONOO^−^) [17,85]. It seems that metformin can regulate these pathways and restore normal conditions in GEC.

S. Takehiko et al. found that metformin may also directly restore the function of eNOS and thereby of GEC. They examined obese Zucker rats, who were administered a placebo or 300 mg/kg/day metformin for 4 weeks. Then, they measured NO concentration [NO] and ONOO^−^ concentration [ONOO^−^] in glomerular endothelial cells from Zucker rats in vitro. Compared with the non-diabetic, glomerular endothelial release of NO was reduced by 41%, while the release of ONOO^−^ increased by 69%. Metformin treatment increased NO concentration by 57%, while decreasing ONOO^−^ by 34%, compared with placebo-treated animals. Treatment with metformin significantly restored the balance in the [NO]/[ONOO^−^] ratio, with a 138% increase for glomerular endothelial cells, whereas fasting glucose level was not meaningfully changed. These findings indicate that metformin therapy has a direct and beneficial effect on GEC function in obese rats, including enhanced NO release and reduced nitroxidative stress, beyond any effects on fasting glucose levels.

It seems that metformin also may reduce the TGFβ1/ALK1-induced renal angiogenesis, which may lead to DKD progression [86].

### 2.4. Podocyte Alterations

#### 2.4.1. Podocytes Alterations in DKD—Overview

**Figure 2:**
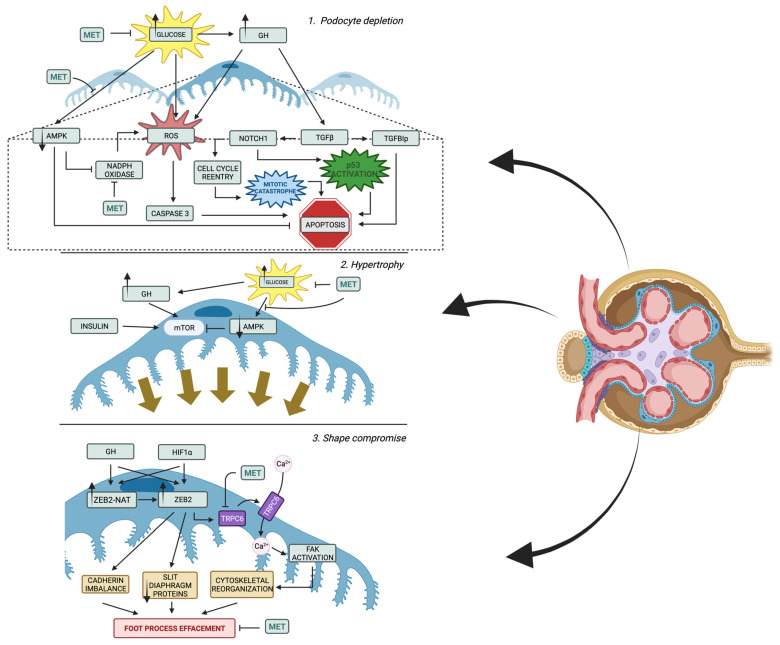
Molecular pathways involved in podocyte alterations in DKD. **1.** Apoptosis of podocytes may be the result of high glucose itself by stimulating reactive oxygen species (ROS) production, but the disrupted inhibiting pathway, including Adenosine Monophosphate-Activated Protein Kinase (AMPK), is also important for its overproduction and, consequentially, caspase 3 stimulation. Podocyte loss can occur also through apoptosis triggered by growth hormone (GH) stimulation. GH induces ROS buildup and higher expression of transforming growth factor-beta (TGFβ). TGFβ leads to the expression of Transforming Growth Factor-Beta-Induced Protein (TGFBIp), which promotes apoptosis. Notch1 activation by TGFβ induces podocyte cell death by activating p53 and Cdk1a. Additionally, podocytes in DKD undergo transition from a dormant state to cell-cycle re-entry, but are unable to complete cytokinesis, resulting in “mitotic catastrophe” and apoptosis. Metformin (MET) does not only influence podocytes’ apoptosis by lowering blood sugar level, but it also activates AMPK and decreases ROS production by inhibiting NADPH oxidase function. **2.** Hypertrophy of podocytes is orchestrated by Mammalian Target of Rapamycin (mTOR), which might be induced by many factors. AMPK decrease seen in DKD progression is also important, since it is an inhibitor of mTOR and its level normalization may reduce hypertrophy in mTOR-independent manner. Metformin inhibits podocytal hypertrophy by decreasing glycemia and activation of AMPK described in detail below. **3.** Podocytes undergo early adaptations that compromise their shape, leading to foot process effacement and slit diaphragm modulations. GH and hypoxia-inducible factor-1α (HIF1α) induce the expression of zinc finger E-box binding homeobox 2 (ZEB2) and its natural antisense transcript (ZEB2-NAT), promoting ZEB2 translation. ZEB2 alters cadherin expression and decreases expression of slim diaphragm proteins, leading to shape compromise. In hypoxic conditions, the HIF1α/ZEB2/TRPC6 axis is activated, leading to calcium influx, focal adhesion kinase (FAK) activation, and cytoskeletal rearrangements. Moreover, these processes might be slowed down by metformin therapy. Created with Biorender.com.

Podocytes along with fenestrated endothelial cells and the glomerular basement membrane form together the glomerular filtration barrier [87]. Podocytes, as central components of the glomerular filtration barrier, play a key role in the pathogenesis of DKD (Figure 2) [88]. Multiple clinical studies have confirmed the correlation between podocyte loss, proteinuria, and glomerulosclerosis, indicating that podocyte loss is potentially a key factor that contributes to DKD progression [64,89,90]. Morphological alterations in podocytes include effacement, flattening of the foot processes, and loosing by apoptosis or detachment [91]. Additionally, many studies suggest a presence of cross-talk between GEC and podocytes and between GEC and mesangial cells. The major mediators for GEC and podocyte communication are vascular endothelial growth factor (VEGF), angiopoietins, and endothelin-1. In DKD, GEC injury may lead to podocyte damage, while podocyte loss further exacerbates GEC injury, forming a vicious cycle [63]. Mature podocytes are terminally differentiated and have no regenerative capacity contrast to GEC, which potentially makes podocytes the weakest component of the glomerular filter [92,93]. For these reasons, the podocytes have become the focus of research on diabetic kidney disease in the past few years.

Increased hyperglycemia translates to elevated ROS production within podocytes and subsequently leads to apoptosis by stimulating p38 mitogen-activated protein kinase and caspase 3 [94]. Moreover, it induces ROS formation indirectly by an elevation of circulating growth hormone (GH), which might switch on the GH-induced generation of ROS [95,96]; the mechanisms of oxidative stress protection are reduced. In experimental models of diabetes, a reduction in adenosine monophosphate-activated protein kinase (AMPK) is observed [97]. This kinase within podocytes may act as an oxidative stress reducer by blocking NADPH oxidase and apoptosis [98]. Since podocytes cannot be restored, decreased glomerular podocyte density might be a permanent change that marks future disease progression [31].

As the podocyte depletion progresses, the podocytes’ population residue adjusts to cover the naked basement membrane. Podocyte alterations include hypertrophy, shape compromise, impaired protein synthesis, and their loss due to detachment, apoptosis, or epithelial-to-mesenchymal transition (EMT).

One of the early adaptations of podocytes in diabetic kidney is shape compromise emphasized by foot process effacement and slit diaphragm modulations. Among drivers of this process, excessive stimulation with GH and hypoxia are best described. Both GH and hypoxia-inducible factor-1α (HIF1α) induce the expression of zinc finger E-box binding homeobox 2 (ZEB2) and natural antisense transcript specific to ZEB2 (ZEB2-NAT), which further promotes ZEB2 translation [99,100,101]. ZEB2 facilitates shape compromise by alternating cadherin expression with P- and E-cadherin downregulation and N-cadherin upregulation [99,101]. As a result, podocyte injury occurs, which results in foot process effacement, decreased slim diaphragm protein expression, and persistent EMT [102]. What is more, diabetic kidney disease is not only associated with cadherin imbalance, but also the reduction in nephrin expression [103]. The loss of nephrin is a result of overexpression of histone deacetylase 4 (HDAC4) and decreased expression of microRNA-29a (miR-29a) which is observed in the diabetic kidney model [104]. Deacetylation of nephrin by HDAC4 leads to its ubiquitination and degradation [104]. miR-29a acts as a regulator of the aforementioned enzyme and alleviates its detrimental effects [104]. Interestingly, TGF-β is a drive leading towards the downregulation of miR-29a and increased HDAC4 expression [105]. Additionally, the stabilization of HIF1α with cobalt chloride also induces decreased slit diaphragm proteins (nephrin and podocin) expression, which once again shows the great impact of hypoxia in DKD progression [106]. What is more, in hypoxic conditions, HIF1α/ZEB2/TRPC6 axis is switched on [107]. Transient receptor potential cation channel, subfamily C, member 6 (TRPC6) facilitates calcium influx into podocyte and consequently enables focal adhesion kinase (FAK) activation [107]. Altogether, this leads to cytoskeletal rearrangements, foot process effacement, and, subsequently, cell shape compromise [107].

Another feature of podocyte adaptation—cell hypertrophy—is activated by excessive mTORC1 signaling, which also plays role in the dysregulation of podocytes autophagy [31]. Indeed, increased mTORC1 activation leads to the acquisition of glomerular phenotype similar to the one of DKD by non-diabetic mice (podocyte hypertrophy, proteinuria, and GBM thickening) [108]. Interestingly, in mouse models, the function of podocytes might be restored when treated with mTORC1 inhibitor—rapamycin [108]. mTOR pathway is activated by many triggers, including GF and insulin [102,109]. In addition, mTORC1 is inhibited by AMPK, the levels of which, as mentioned above, are reduced in experimental models of diabetes [97,110]. Although, in the study by Banu et al., the increase in the activation of AMPK itself, rather than mTOR modulation, was a driver of podocyte size normalization [111]. Therefore, the enlargement of podocytes is probably a result of many intertwined molecular pathways that need further clarification.

The loss of podocytes occurs in several manners. Firstly, the stimulation of podocytes by GH stimulates their production of GBM components in streptozotocin-diabetic rats and in vitro models [112,113,114]. In addition, the GF stimulation is correlated with the decreased expression of matrix metalloproteases (MMPs), which degrade extracellular matrix proteins [112,114]. As a result, the thickening of GBM occurs, which results in the impaired adhesion of podocytes and their detachment. Secondly, the loss of podocytes might happen due to apoptosis. Interestingly, there is a growing body of knowledge highlighting the importance of GH stimulation as the trigger of this process [102]. As mentioned above, ROS overproduction is often associated with apoptosis and GH may trigger ROS buildup [96,102]. Additionally, GH-induced podocytes have higher expression of TGFβ, which drives podocyte depletion in several ways [115,116]. Apoptosis occurs when podocytes express Transforming Growth Factor-Beta-Induced Protein (TGFBIp) due to GH stimulation [112]. Moreover, TGFβ induces Notch1, which subsequently activates p53 and Cdk1a, prompting cell death and glomerulosclerosis [117]. The Notch1-induced podocyte cell death is a complicated process that involves podocyte transition from a dormant state to the cell-cycle re-entry when stimulated with GH or TGFβ [115]. Podocytes, which are terminally differentiated cells, despite undergoing karyokinesis, are not able to complete cytokinesis, which activates so-called “mitotic catastrophe” and, as a result, apoptosis [115]. Lastly, podocytes might also be lost in a process called epithelial-to-mesenchymal transition, which also may be triggered by GH overstimulation [118]. The process is mediated by TGF-β, which is overexpressed in DKD [108]. Podocytes incubated with TGF-β change their protein expression with slit diaphragm-associated proteins’ expression decrease (P-cadherin, zonula occludens-1, and nephrin) and collagen I, desmin, fibronectin, and matrix metalloproteinase-9 expression increase. Altogether, these adjustments translate to increased albumin permeability [109]. As mentioned before, TGF-β is an activator of Notch signaling, which is also an important feature in podocyte differentiating [110].

#### 2.4.2. Impact of Metformin on Podocytes

- Regulation of glucose uptake and reduction in insulin resistance by metformin

Phosphatase and tensin homolog (PTEN) is a dual-function lipid and protein phosphatase that serves as an important negative modulator of the insulin signaling pathway [119]. D. Rogacka et al. showed that long-term exposure of podocytes to hyperglycemic conditions attenuates the stimulatory effect of insulin on glucose uptake into podocytes via the AMPK (AMP-activated protein kinase) –PTEN pathway and also the high level of glucose decreases AMPK phosphorylation, thereby increasing PTEN protein levels, attenuating the insulin responsiveness of podocytes. Metformin caused AMPK activation in both control and hyperglycemic podocytes and stimulated glucose uptake and significantly decreased PTEN protein levels in podocytes cultivated under control and high-glucose conditions. This study also used an AMPK inhibitor to attenuate insulin-dependent glucose uptake, suggesting that AMPK is essential for insulin activity in podocytes. PTEN protein level was also associated with the modulation of AMPK activity, which suggests it increases after AMPK inhibition in podocytes cultured under normoglycemic conditions [120].

Another target of metformin in podocytes in the context of regulating glucose uptake and insulin responsiveness is Sirtuin-1 (SIRT1), which is a nicotinamide adenine dinucleotide (NAD)-dependent protein deacetylase. It is a crucial regulator of glucose and lipid metabolism through direct or indirect involvement in insulin signaling [121]. The levels of AMPK and SIRT1 are measurably decreased in the diabetic kidney animal models, which correlate with higher amounts of reactive oxygen species (ROS) and increased apoptosis, inflammation, and renal damage. Moreover, stimulation of the AMPK/SIRT1 signaling pathway is associated with protection against high glucose-induced renal injury [93].

D. Rogacka et al. observed a stimulating effect of metformin on the enzymatic activity of SIRT1 in podocytes. They demonstrated that high glucose concentrations do not alter metformin’s ability to activate SIRT1 in podocytes. Metformin also prevented the hyperglycemia-induced reduction in SIRT1 protein level in these cells. In parallel, metformin increased AMPK phosphorylation in podocytes incubated in control and hyperglycemic medium. As a result, hyperglycemic-induced impairment of glucose uptake into podocytes was ameliorated [122]. These observations suggest that metformin simultaneously activates SIRT1 and AMPK and may improve the insulin responsiveness of podocytes, preventing diabetic complications [93,122].

Another negative regulator of insulin is SH2-domain-containing inositol polyphosphate 5-phosphatase 2 (SHIP2) [123]. SHIP2 dephosphorylates PIP3, affecting PIP3 levels and downregulating signaling induced, among others, by insulin [124]. Overexpression of SHIP2 in podocytes reduces Akt activation in response to insulin and promotes apoptosis. M. E. Hyvönen et al. in a study on rats found that SHIP2 is an important negative regulator of insulin signaling in podocytes [125]. Interestingly, Polianskyte-Prause et al. demonstrated that metformin was also shown to exert actions on glucose uptake in podocytes via reduced SHIP2 activity. They also reported that SHIP2 reduces Akt activity and enhances podocyte apoptosis, and both are restored by metformin to control levels [126].

- Prevention of slit diaphragm injury by metformin

Podocytes’ cell body connects with the GBM through major processes with long interdigitating foot processes. Mature podocytes form specialized cell–cell junctions between interdigitating foot processes—slit diaphragms—and represent the main filter barrier in the kidneys. Slit diaphragms include of P-cadherin, tight junction protein-1 (ZO-1), transient receptor potential channel 6(TRPC6), actin, nephrin, podocin, and Neph1, of which podocalyxin is a transmembrane sialoglycoprotein that is essential for maintaining the structure of interdigitating foot processes and nephrin is the most important molecule in intracellular signaling including insulin responsiveness of podocytes [93,127].

M. Szrejder et al. found that the diabetic condition reduced nephrin expression in cultured rat podocytes. They also showed that metformin treatment restored nephrin expression and increased the amount of nephrin detected close to the cell surface in podocytes exposed to high glucose. Moreover, increased urinary nephrin levels could indicate the degree of podocyte damage and be a urinary marker of podocytopathy [128].

L. Zhai et al. showed a similar conclusion in their examination of T2DM rats treated with metformin. They demonstrated that metformin produces significant reductions in urinary albumin and nephrin concentrations, glomerular basement membrane thickness, and the foot process fusion rate compared with control T2DM model rats. Moreover, renal expression of nephrin protein and Nphs1mRNA was increased by metformin treatment. This results in the conclusion that metformin protects kidney podocytes in T2DM model rats by adjusting renal nephrin expression [129].

Moreover, M. Szrejder’s study revealed that the effect of metformin on nephrin protein levels in podocytes is AMPK-dependent because of the fact that AMPK inhibitor, compound C, decreased nephrin protein levels in normoglycemic (control) podocytes to values observed in podocytes cultivated in hyperglycemia [128].

Additionally, L. Zhai et al. examined metformin’s impact on podocalyxin (PCX). They studied the effect of metformin treatment on renal tissue PCX expression in type 2 diabetic rats (T2DM was induced by high-fat diet/streptozotocin (HFD-STZ)) and clarified its protection on glomerular podocytes. Diabetes induced significant alterations in renal glomerular structure, increased albumin and PCX levels in urine, and highly decreased PCX protein and mRNA expression of renal tissue. However, metformin treatment restored all these changes to a varying, dose-dependent degree. These results suggested that metformin can ameliorate glomerular podocyte damage, which may be associated with its role in restoring PCX expression and inhibiting urinary excretion of PCX [130].

- Modulation of podocyte cytoskeleton and improvement of filtration barrier permeability by metformin

The rearrangement of proteins expressed in podocytes can modify the surface area for filtration [93]. As was mentioned, the foot processes of neighboring podocytes interact to establish a highly branched, interdigitating system, known as the slit diaphragm [131]. The slit diaphragm represents a signaling platform with many proteins that regulate podocyte function, including nephrin, podocin, Neph1, CD2AP, TRPC6, BK_Ca_, and actin [128]. The Rho-family small guanosine triphosphatases (GTPases) also play a crucial role in cytoskeleton organization in podocytes. An early occurrence during the development of diabetic nephropathy is cytoskeletal rearrangement, which may alter the integrity of slit diaphragms, resulting in albuminuria and DKD progression [93].

As mentioned earlier study, M. Szrejder et al. demonstrated that AMPK activation was necessary for maintaining appropriate levels of Rho-family GTPase activity in hyperglycemic conditions. In their examination, metformin through AMPK activation remodeled cytoskeleton dynamics and, consequently, reduced filtration barrier permeability in diabetic conditions. Moreover, metformin normalized TRPC6 expression via AMPKα1 activation in podocytes exposed to hyperglycemia [128]. Another study revealed an important correlation between high Ca^2+^ levels and podocyte injury. The authors found that TRPC6 overexpression downregulates nephrin and synaptopodin expression and stimulates RhoA activity, leading to F-actin rearrangement and a reduced number of foot processes [132]. In respect of Szrejder’s study, metformin appears to normalize this signaling pathway by reducing TRPC6 expression in podocytes in hyperglycemia.

Additionally, in the latest study, LP. Kolligundla et al. investigated the regulation of transglutaminase 2 (TG2) by HIF1α in podocytes, essential for the glomerular filtration barrier. HIF1α induced TG2 expression and activity through the ZEB2/TRPC6 axis, leading to increased intracellular calcium levels. Metformin prevented HIF1α-induced podocyte injury and proteinuria, suggesting its potential as a treatment in chronic kidney disease [133].

Furthermore, as mentioned above, D. Rogacka et al. demonstrated that metformin, besides its impact on glucose uptake into podocytes, can decrease glomerular filtration barrier permeability by activation of SIRT1 and AMPK and prevention of hyperglycemia-induced reduction in SIRT1 protein levels [122]. It was shown that SIRT1 can play a role in maintaining the actin cytoskeleton in podocytes [134]. However, this experiment was conducted in normoglycemic conditions, so it has to be checked in a study dedicated to DKD.

- Buffering oxidative stress, activation autophagy, and suffering from apoptosis by metformin

It is posited that localized tissue oxidative stress is a key component in the development of diabetic nephropathy [135]. High intracellular ROS levels are prospective inducers of apoptosis in podocytes and this correlates with increasing albuminuria [94].

J. Kim et al. examined the protective effects of metformin on the injury of renal podocytes in spontaneously diabetic Torii (SDT) rats, a model for non-obese type 2 diabetes. Diabetes induced significant alterations in renal glomerular structure, which was expressed in highly increased urinary and renal 8-OHdG levels and podocyte loss. They showed that metformin normalizes the level of the oxidative stress marker 8-hydroxydeoxyguanosine (8-OHdG) in both urine and kidney tissue. Notably, metformin reduced the level of 8-OHdG, especially in podocytes, which is associated with reduced podocyte loss by apoptosis. Interestingly, in this study, metformin only subtly decreased blood glucose and HbA1c in rats’ blood, indicating that the protection of podocytes from apoptosis is independent of lowering hyperglycemia [136].

In another study, A. Piwkowska et al. investigated the effect of metformin on the production of superoxide anion in cultured podocytes. Superoxide anion is produced by NAD(P)H oxidase and is the main source of ROS in diabetic podocytes. Authors showed that metformin reduces the high glucose-induced excessive activity of NAD(P)H oxidase and, thereby, ROS production in podocytes [137].

The same team in another study revealed that metformin increased extracellular ATP concentration by reduction in ecto-ATPase activity, decreased NAD(P)H oxidase activity, and increased AMPK phosphorylation. Similar effects are observed after the activation of P2 receptors. Moreover, when the activation of P2 receptors was prevented by the P2 receptor antagonist (suramin), the inhibition effect of metformin on AMPK and NAD(P)H oxidase activation also was abolished. The results suggest that some effects of metformin may be mediated via activating the purinergic P2 receptors [138].

R. Huiwen et al. explored the molecular mechanism of metformin alleviating DKD via AMPK/SIRT1-FoxO1 pathway by measuring the levels of oxidative stress and autophagy-related factors in diabetic rats with high-fat diet and low-dose streptozotocin (STZ). They found that metformin efficiently buffered the renal function injury in diabetic rats and relieved oxidative stress, enhancing autophagy through AMPK/SIRT1-FoxO1 pathway [139].

J. Xu et al. also studied the Sirt1/FoxO1 autophagic signaling pathway and induction of renal autophagy. The results showed that metformin could protect renal function by upregulating autophagy levels and alleviating oxidative stress levels of renal tissue and pathological and structural changes in glomeruli. They also used Sirt1 inhibitor EX527 and metformin to observe whether the protective effect of metformin was achieved through the Sirt1/FoxO1 autophagic signaling pathway. EX527 could block the protective effect of metformin on diabetic rats’ kidneys, which suggests that metformin could extenuate kidney injury in diabetic rats by inducing the Sirt1/FoxO1 autophagy signal axis [140].

Neither of these two studies above addressed whether metformin enhances autophagy specifically in podocytes but looked at autophagy in the kidney in general.

The high glucose level also modulates AMPK signaling and cell apoptosis. S. Langer et al. investigated the effect of metformin on podocytes’ apoptosis and pro- and antiapoptotic signaling via AMPK and mammalian target of rapamycin (mTOR) activation in these cells. They showed that metformin was abolished and reversed the high-glucose-induced reduction in AMPK phosphorylation and induction of mTOR in podocytes. Furthermore, metformin reduced high-glucose-induced podocyte apoptosis. These results indicate that metformin has an antiapoptotic impact on podocytes under high-glucose conditions via activation of AMPK and inhibition of mTOR signaling [141].

### 2.5. Mesangial Cell (MC) Injury

Mesangial expansion is one of the most pronounced histological findings in diabetic patients [142]. Indeed, MCs also suffer from intracellular alterations due to high glucose levels and are of great importance in the pathobiology of DKD.

As mentioned, TGF-β plays a role in EndoMT and EMT, inducing glomerular fibrosis. This agent is also overexpressed in MCs stimulated by hyperglycemic conditions [143]. Although, other diabetes-related traits such as blood glucose fluctuation and increased AGEs production can also lead to greater TGF-β induction [144,145]. TGF-β pathway activation within MCs is pronounced by its synthetic phenotype, increased growth, and elevated extracellular matrix (ECM) [146]. What is more, it enhances glucose transporter 1 (GLUT1) expression within MC, which translates into increased glucose uptake and, thus, may be a starting point for disturbed intracellular metabolism and its negative effects [147].

What is more, other molecular pathways, namely Wnt/β-catenin, mediate pathogenic repair in diabetic MCs [31]. The decrease in Wnt4 and Wnt5a signaling within MCs stimulated with high glucose corresponds with increased glycogen synthase kinase-3β (GSK-3β) and caspase 3 activation, which translates to mesangial injury [148]. This effect is partially caused by superoxide build-up and following Ras/Rac1 induction [148]. In line with this finding, inhibiting both Ras and Rac1 decreases apoptotic cell count and reverses impaired Wnt5a/β-catenin signaling [148]. Interestingly, increased Ras activation is not only seen after high glucose stimulation, but is also pronounced after conditioning with AGEs [149]. Altogether, Ras increases ERK and c-Jun pathways, which create complex cross-talks with TGF-β, inducing glomerular sclerosis [150]. Moreover, hyperglycemia induces the expression of Dickkopf-1 (DKK1), which inhibits the Wnt/β-catenin pathway [151]. An introduction of DKK1 cDNA induces the expression of profibrotic factors such as TGF-β and fibronectin in cell cultures [151]. Additionally, treatment with DKK-1 antisense oligonucleotides decreases the excretion of urinary protein in vivo [151].

### 2.6. Inflammation and Fibrosis

#### 2.6.1. Inflammation and Fibrosis in DKD—Overview

A thorough review of inflammation and renal fibrosis is beyond the scope of this paper and has been described elsewhere [150,152,153]. However, a brief introduction to the major pathological events is provided to ensure a better understanding of the metformin effects on these traits of DKD.

One of the crucial actions within the kidney which sets the milieu for further fibrosis is inflammation. Hyperglycemia and other metabolic alterations induce activation of the mononuclear phagocyte system within a kidney, which translates to cytokine release, increased infiltration of monocytes and macrophages, and, finally, exacerbated morphological alterations in the glomerulus [154]. A recent study by Nishad et al. has demonstrated that podocytes stimulated by GH activate TNF-α signaling, which induces differentiation of monocytes to macrophages and, consequently, proteinuria, renal inflammation, and fibrosis [155]. In line with that, the macrophage accumulation within a diabetic kidney corresponds with the decline in renal function [156]. Other inflammatory cells that seem to be important in the pathogenesis of fibrosis are dendritic and mast cells [152]. Not only inflammatory cells but also renal cells are able to produce numerous cytokines (e.g., tumor necrosis factor alpha (TNFα), interleukin (IL)-1, IL-6, and IL-17), chemokines (e.g., monocyte-chemotactic protein-1 (MCP-1)/chemokine C-C motif ligand 2 (CCL2)), and other molecules that intensify the inflammatory process and tissue damage [154].

Chronic inflammation and injury in diabetic kidneys lead to the activation of ECM-producing cells—myofibroblasts [152]. As described above, the mesenchymal transition from endothelial cells or podocytes is possible through TGF-β stimulation. However, there are other cell populations that are a source of myofibroblasts within a kidney: resident fibroblasts, bone marrow (BM)-derived fibrocytes, and pericytes [153]. The process of transition from these cells to activated myofibroblast is also driven by TGF-β [150]. Disregarding the cellular source of myofibroblasts, when activated, they produce ECM components such as fibronectin, procollagen, vitronectin, and thrombospondin [152]. Firstly, the deposited ECM is prone to being degraded but, with the fibrosis progression, the accumulated matrix causes irreversible kidney damage [152].

#### 2.6.2. Impact of Metformin on Inflammation and Fibrosis in DKD

Macrophage migration inhibitory factor (MIF) is increased in the kidney and urine in DKD. Activation of CD74 (a receptor for MIF) by MIF leads to an inflammatory response in podocytes. CD74 is expressed in podocytes and its expression is upregulated in DKD [157].

Y. Xing et al. observed the effects of metformin on the urinary excretion of MIF, CD74, and podocalyxin in people with T2DM. They compared the amount of MIF and CD74 in serum, urinary MIF-to-creatine ratio (UMCR), urinary CD74-to-creatine ratio (UCCR), urinary albumin-to-creatine ratio (UACR), and urinary podocalyxin-to-creatine ratio (UPCR) in patients receiving either sulfonylurea (*n* = 100) or sulfonylurea and metformin (*n* = 102) for 24 weeks. The study showed that metformin add-on therapy reduces UMCR, UCCR, UPCR, and UACR in comparison with those in the sulfonylurea monotherapy group. The authors conclude that metformin reduces the inflammatory signaling by MIF–CD74 in podocytes and could, thus, protect the podocytes from injury. This effect of metformin is independent of the glycemic control because there were no differences in the fasting blood glucose of HbA1c values between the examined groups [158]. This can confirm that metformin has an anti-inflammatory effect but it should be checked in a larger study with modern antidiabetic drugs.

## 3. Conclusions

In the view of high heterogeneity of DKD and the lack of direct pharmacotherapy, we need to slow down the progression of this disease with available drugs. Hyperglycemia activates numerous molecular pathways which induce irreversible changes in the glomeruli. However, most of these pathways can be regulated by metformin. This old, well-known drug reduces ROS production, which attenuates the negative impact of increased glucose levels on kidneys. Moreover, this drug has a protective effect that is revealed in podocytes and endothelial cells among diabetic patients and animals. However, it is worth mentioning that it is still not well determined whether the positive effect of metformin therapy is mediated directly by metformin or indirectly by reduction in hyperglycemia. An understanding of the nephroprotective mechanism of metformin may help in new, direct therapy design. It seems that “omics” studies (genomics, transcriptomics, proteomics, and metabolomics) and a better understanding of pathogenesis will be essential in this progress.

## Figures and Tables

**Table 1:** Main metabolic pathways induced by hyperglycemia. AGE—advanced glycation end products; DAG—diacylglycerol; DKD—diabetic kidney disease; PKC—protein kinase C; ROS—reactive oxygen species; UDP-GlcNAc—uridine diphosphate-N-acetylglucosamine.

Pathway	Main Substrate	Main Product(s)	Cellular Alterations
Polyol pathway	glucose	sorbitol; fructose	- induction of osmotic and oxidative stress
			- increased usage of NADPH
Hexosaminepathway	fructose-6-phosphate	UDP-GlcNAc	- alterations in gene expression
			- increased ROS production
			- increased caspase-3 activity
PKC pathway	dihydroxyacetonephosphate	DAG	- activation of PKC
			- increased ROS production
			- decreased nitric oxide
AGE pathway	variable: proteins, glucose, amino acids, metabolites	AGE	- increased ROS production
			- activation of inflammatory response
			- decreased nitric oxide and taurine production

## Data Availability

Not applicable.
